# Phosphorylation of eukaryotic initiation factor eIFiso4E enhances the binding rates to VPg of turnip mosaic virus

**DOI:** 10.1371/journal.pone.0259688

**Published:** 2021-11-04

**Authors:** Mateen A. Khan, Pankaj Kumar, Mohd. Akif, Hiroshi Miyoshi

**Affiliations:** 1 Department of Life Science, College of Science and General Studies, Alfaisal University, Riyadh, Saudi Arabia; 2 Department of Biochemistry, School of Life Science, University of Hyderabad, Hyderabad, India; 3 Department of Microbiology, St. Marianna University School of Medicine, Kawasaki, Japan; George Washington University, UNITED STATES

## Abstract

Binding of phosphorylated eIFiso4E with viral genome-linked protein (VPg) of turnip mosaic virus was examined by stopped-flow, fluorescence, circular dichroism (CD) spectroscopy, and molecular docking analysis. Phosphorylation of eIFiso4E increased (4-fold) the binding rates as compared to unphosphorylated eIFiso4E with VPg. Stopped-flow kinetic studies of phosphorylated eIFiso4E with VPg showed a concentration-independent conformational change. The dissociation rate was about 3-fold slower for eIFiso4E∙VPg complex upon phosphorylation. Phosphorylation enhanced the association rates and lowered the dissociation rates for the eIFiso4E∙VPg binding, with having higher preferential binding to eIFiso4Ep. Binding rates for the interaction of eIFiso4Ep with VPg increased (6-fold) with an increase in temperature, 278 K to 298 K. The activation energies for binding of eIFiso4Ep and eIFiso4E with VPg were 37.2 ± 2.8 and 52.6 ± 3.6 kJ/mol, respectively. Phosphorylation decreased the activation energy for the binding of eIFiso4E to VPg. The reduced energy barrier suggests more stable platform for eIFiso4Ep∙VPg initiation complex formation, which was further supported by molecular docking analysis. Moreover, far-UV CD studies revealed that VPg formed complex with eIFiso4Ep with substantial change in the secondary structure. These results suggested that phosphorylation, not only reduced the energy barrier and dissociation rate but also enhanced binding rate, and an overall conformational change, which provides a more stable platform for efficient viral translation.

## Introduction

Viruses depend on the translational apparatus of the host cells and have developed sophisticated mechanisms to suppress translation of cellular mRNAs whereas ensuring its own translation. Many viruses use a 5’-cap-dependent mechanism, while other viruses use a cap-independent mechanism for initiation. Turnip mosaic virus (TuMV) is a positive strand RNA virus belonging to the *potyviridae* family [1]. While *potyviridae* mRNAs possess poly(A) tail at their 3’-terminus, they lack m^7^G cap structures and instead carry viral genome-linked proteins (VPgs) that are covalently attached to the 5’-terminus [2]. VPg of *Potyvirus* mRNA plays an important role in cap-independent translation mechanism. There are multiple suggested roles of VPg in initiation of viral protein synthesis. It functions as a substitute of eukaryotic mRNA cap moiety structure, recruiting 40S ribosomal subunits and initiation factors to viral mRNA in preference to cellular mRNA [3, 4]. Interaction of VPg with eukaryotic initiation factor 4E (eIF4E) is essential for viral infection [5–7]. VPg plays important role for the initiation of *calicivirus* protein synthesis. Removal of VPg from feline *calicivirus* RNA results in loss of infectivity and dramatically reduces translation [8, 9]. Interactions of VPg with the viral RNA polymerase in yeast support a role in viral RNA synthesis [10]. VPg of potato virus A, tobacco etch virus, pea seed-borne mosaic virus, and turnip mosaic virus were shown to determine host specificity for systemic movement or replication [11–14]. Interaction of VPg with eukaryotic initiation factors has been investigated by various biochemical and biophysical methods [15–18]. X-ray structure and nuclear magnetic resonance studies showed that the eIF4E-VPg and eIF4E-mRNA cap complexes are stabilized through hydrogen bond formation [17, 19]. Protein-protein interactions and conformational changes of proteins play an important role in many biological processes and are required for host-pathogen communication [20, 21]. VPg has also been implicated for vascular movement of the virus and more directly implicated by mutagenesis, in long distance translocation of the virus [22]. Moreover, VPg has been involved in overcoming the avirulence factor for recessive resistance genes in many plants [11, 12].

Translation regulation of protein synthesis requires a series of events that allow the recruitment of multiple initiation factors. In eukaryotes, mRNA translation initiation occurs through a cap-dependent mechanism where an m^7^G mRNA cap is recognized by the eIF4F translation factor [23]. The eIF4F translation factor is composed of the eIF4E cap-binding protein, the eIF4G scaffolding protein, and the eIF4A RNA helicase [24]. In plants, there is a second eIFiso4F complex, which is composed of eIFiso4E and eIFiso4G [25]. EIF4F binding to the cap and PABP binding to the poly-(A) tail circularizes to activate the mRNA. The 43S pre-initiation complex then binds near the cap and scans the 5’-untranslated regions for an AUG codon [23]. Recognition of start codon comprises the first stage of the cellular mRNA translation. EIF4G in turn recruits additional initiation factors, which unwinds secondary structure within the 5’-leader sequence to allow 40S ribosomal scanning and promote mRNA translation [26]. In contrast to the eukaryotic translation mechanism, viral mRNA utilizes VPg or an internal ribosome entry site for translation initiation. The eIF4E or eIFiso4E complexes found in plants are key interacting partners of the VPg proteins found in many RNA viruses and are required for infection [7, 27]. Physical interaction between VPg of potyviruses with eIF4E has been demonstrated in multiple plant-potyvirus systems. For example, VPg of lettuce mosaic virus, potato virus Y, sapo virus, and TuMV interact with eIF4E or its isoform eIFiso4E and increases viral infectious ability *in vivo* [28, 29]. Interaction of biological macromolecules VPg to eIF4E or its isoform eIFiso4E induces structural changes of the cap binding protein and diminishes the cap binding ability of initiation factors, resulting in the interruption of the formation of the translation initiation complex [15, 17]. Structural changes of initiation factors binding to VPg or eIF4E∙VPg complexes have been investigated by several biophysical techniques such as circular dichroism and 3-dimensional structure [17, 18, 30, 31]. However, VPg induced conformational changes of phosphorylated eIFiso4E (eIFiso4Ep) are unknown. Phosphorylation of translation initiation factors plays an important role for interactions between initiation factors and RNA. Initiation factors are phosphorylated during development and stress conditions such as heat shock or hypoxia [32, 33]. Viruses manipulate the host cell translational machinery by phosphorylation of eIF4E. EIF4E has been shown to be phosphorylated following infections of multiple viruses such as murine norovirus, herpes simplex virus, human cytomegalovirus [34]. Phosphorylation increased the translation by higher binding affinity of eIF4E with mRNA cap [35]. These observations suggested that phosphorylation of eIF4E is crucial for regulatory translational mechanism, which might mediate the functional interaction between viral VPg and host plant isoform eIFiso4E. We have previously [36] investigated that phosphorylation of eIFiso4E enhances the binding of eIFiso4E∙VPg and mRNA translation *in vitro*. To further examine the role of phosphorylation, we used stopped-flow and circular dichroism to dissect the kinetic mechanism and structural changes of eIFiso4Ep binding to VPg.

## Materials and methods

### Materials

HiTrap chromatography columns (Mono Q) was purchased from GE Healthcare. Sephadex G-25 was acquired from Pharmacia Fine Chemicals Inc. Piscataway, New Jersey. DEAE Cellulose was obtained from Whatman International Ltd. Maidstone England. m^7^GTP-Sepharose-4B was purchased from Amersham Biosciences Co. Ltd., and His-bind Ni-resin was purchased from Novagen Co. Ltd. USA.

### Purification of proteins

Recombinant wheat eIFiso4E was expressed in *Escherichia coli* containing the constructed pET3d vector in BL21-(DE3)-pLysS as described previously [37]. After overnight induction at 293 K, pelleted cells were resuspended in 20 mM HEPES/KOH, pH 7.6, 1 mM DTT, 0.1 mM EDTA, 600 mM KCl, 0.1% triton x-100 containing 0.5 mL aprotinin, and 100 μg/ml trypsin inhibitor. Cells were lysed by sonication. A Hitrap Mono Q ion exchange column was used for the purification of protein. Eluted fractions were mixed with pre-equilibrated m^7^-GTP-sepharose-4B beads for affinity purification. The expressed eIFiso4E was eluted by 100 mM GTP. Purity of eIFiso4E protein was analyzed by running 12.5% sodium dodecyl sulfate-polyacrylamide gel electrophoresis (SDS-PAGE), and high levels of purity (>95%) were obtained.

The TuMV cDNA clone and construction of the expression vector for VPg was described previously [15, 38]. VPg was expressed in *E*. *coli* containing the constructed pET3d vector in BL21-(DE3)-pLysS as described elsewhere [3, 38]. A nickel column, mono Q column, and Hiload 16/60 superdex 75 pg columns were used for the purification of VPg. The purified VPg fractions were pooled and concentrated in a Centricon-10 filter. Purity of VPg protein was confirmed by running 15% SDS-PAGE. Protein bands were stained with Coomassie Blue.

Recombinant eIFiso4E was phosphorylated *in vitro* of as described elsewhere [33, 36]. Five hundred μg eIFiso4E was incubated at 303 K for 2.5 hour in a 1.0 ml reaction mixture containing casein kinase II (100 unit), 500 μM ATP, 10 mM MgCl_2_, 100 mM NaCl, 1 mM dithiothreitol, and 0.1 mM EDTA in 20 mM Tris-HCl, pH 7.0 with continuous mixing. Phosphorylated eIFiso4E was confirmed by HPLC as reported previously [39] and further verified by native polyacrylamide gel electrophoresis.

Protein samples were dialyzed against titration buffer 20 mM HEPES/KOH, pH 7.6, containing 150 mM KCl, 1.0 mM MgCl_2_ and 1.0 mM DTT, and passed through a 0.22 μm filter (Millipore) before the spectroscopy measurement. Protein concentrations were determined by the Bradford method [40] using Bio-Rad protein assay reagent with bovine serum albumin as standard.

### Stopped-flow kinetics measurements

Rapid kinetic measurements for the binding of eIFiso4Ep with VPg of turnip mosaic virus were performed with a stopped-flow spectrometer. The dead time of the instrument was 1 milli-second. Fluorescence intensity (measured in volts) for the eIFiso4Ep was monitored at the cut-on filter of 324 nm with an excitation wavelength of 280 nm. Fluorescence intensity was monitored up to 200 ms. About 1000 pairs of data points were collected in each sample reaction. The samples were incubated for 15 minutes as needed to allow to equilibrate to experimental temperature prior to the data collection. To determine the temperature-dependent rate constants, the sample was thermo-stated, and the temperature of the flow-cell reservoir was maintained with a temperature-controlled circulating water bath (ΔT ± 273.25 K). After rapid mixing of the eIFiso4Ep with VPg, the time course of the fluorescence intensity was recorded by computer data acquisition. The concentration of eIFiso4Ep in one syringe was 0.2 μM (0.1 μM after mixing). The concentration of VPg in the other syringe was 2 μM (1 μM after mixing). Samples were passed through 0.22 μm filter and degassed prior to loading into the syringe. The stopped-flow traces are the average of three-to-five individual shots to improve the signal-to-noise ratio. Each averaged set of stopped-flow fluorescence data was then fitted to nonlinear analytical equations using GlobalWorks^TM^ analysis software.

### Kinetic data analysis and curve fitting

Stopped-flow fluorescence data were used to determine the rate constant value for the binding of eIFiso4Ep with VPg of turnip mosaic virus. Data was analyzed according to a curve-fitting program, Global analysis software as described previously [4, 41]. Data were fit to the single- and double-exponential functions. Fitted curves correspond to the following single exponential equation,

$$F_{t}=R\times e^{\left(-k_{obs}\times t\right)}+F_{f}$$ (Eq 1)

where *k*_obs_ and *R* are the observed first-order rate constant and amplitude, respectively. *F*_*f*_, is the final value of fluorescence, and *F*_t_ is the fluorescence observed at any time, *t*. Fitted curve correspond to the following double-exponential equation,

$$F_{t}=R_{1}\times e^{\left(-k_{{obs}_{1}}\times t\right)}+R_{2}\times e^{\left(-k_{{obs}_{2}}\times t\right)}+F_{f}$$ (Eq 2)

where *R*_1_ and *R*_*2*_ are the amplitudes of the two exponentials, and *k*_obs1_ and *k*_obs2_ are the observed rate constants for the first and second components of the double-exponential fits. The subscripts 1 and 2 refer to the fast and slow phases, respectively. An assessment of each fit was made from the residuals, which measure the difference between calculated fit and the experimental data. KaleidaGraph software (Version 2.1.3, Abelbeck software) was used for least-square fitting of data with linear equation and determination of standard errors for parameters obtained from the fits. The rate constant values are represented as the mean standard deviation (± SD).

To determine the activation energy of the complex eIFiso4Ep with VPg of TuMV, temperature-dependent observed rate constants were used to construct Arrhenius plots according to the equation,

$$\ln\;k_{obs}=(\frac{E_{a}}{RT})+\ln\;A$$ (Eq 3)

where *k*_*obs*_ is the observed rate constant, *E*_a_ is the activation energy, T is the absolute temperature, *R* is the universal gas constant, and *A* is the Arrhenius pre-exponential factor. The value of *E*_a_ was calculated using the slopes of the fitted linear plot of ln *k*_*obs*_ *versus* T^-1^ (Kelvin).

### Dissociation rate constants measurements

Dissociation rate constant of the pre-formed eIFiso4Ep∙VPg and eIFiso4E∙VPg complexes were measured by spectrofluorometer equipped with excitation and emission polarizers. Fluorescence intensity values for each sample were monitored at an excitation wavelength of 280 nm and emission wavelength of 340 nm. The excitation and emission slits were set at 4 and 5 nm, respectively. The excitation slits were chosen to avoid photo-bleaching, and the absorbance of the sample at the excitation wavelength was less than 0.02 to minimize the inner-filter effect. eIFiso4Ep∙VPg and eIFiso4E∙VPg complexes were incubated in titration buffer for about 15 minutes to ensure complex formation at 298 K. To measure the dissociation rate constants of eIFiso4Ep∙VPg and eIFiso4E∙VPg complexes were rapidly diluted to 10-fold with titration buffer in a spectrofluorometer cuvette, and the resulting increase in fluorescence intensity was measured. Because of the strong interactions between the protein-protein complexes, a large dilution, which could not be accomplished by stopped-flow, was necessary. The concentrations of the reactants after mixing were 10 μM VPg and 1 μM of eIFiso4Ep or eIFiso4E. The dissociation rates were determined from the fits of a single-exponential equation to the data using the nonlinear least-square fitting program Kaleida Graph software.

### Circular Dichroism (CD) spectroscopy

CD measurements were performed on a Chirascan Plus spectropolarimeter (Applied photo physics Ltd, UK) equipped with a thermostatically controlled circulating water bath with cell holder under constant nitrogen flow as described elsewhere [42]. Temperature for each sample was maintained at 298 K. Far-UV CD spectra (200–250 nm) of eIFiso4Ep were obtained in titration buffer. All spectra were obtained at constant eIFiso4Ep concentration (0.1 μM) with a 0.5 mm path-length quartz cell. Each spectrum was collected at the interval of 1.0 nm, bandwidth 1 nm, and scan speed of 100 nm/min with a response time of 2 sec. The instrument was calibrated with (D)-(+)-10-camphorsulfonic acid according to the procedures out lined by the manufacturer. Far-UV CD measurements of 0.1 μM eIFiso4Ep protein were obtained with addition of different amounts of viral genome-linked protein VPg (0–1 μM). The eIFiso4Ep solution was prepared by dialyzing into 20 mM Tris-HCl, pH 7.5. Each spectrum was measured after incubating each protein sample in the absence and presence of VPg for 15 minutes at 298 K. The protein samples for CD measurements were dialyzed and filtered through a Millipore filter (0.22 μm) to remove any suspended material. Each spectrum was comprised of the average of three-to-five scans and high frequency noise reduction applied before final CD spectra. For each spectrum collected, the contribution from the buffer blanks and buffer containing 0–1 μM VPg where applicable, were subtracted from the respective spectra. All the spectral data were processed and averaged using the online server Dichroweb, (http://dichroweb.cryst.bbk.ac.uk/html/links.shtml) [43]. Secondary structural contents were also estimated from the amino acid sequence by the predictive methods as described previously [44, 45]. Helical content of the protein was determined by Dichroweb agrees well with the value estimated from the mean residue ellipticity (MRE) at 222 nm using the following equation [44, 46]:

$$\%\;\alpha -\mathrm{helix}=(\frac{{MRE}_{222nm}-2340}{30300})\;X\;100$$ (Eq 4)

### Molecular modeling and docking

The VPg sequence of turnip mosaic virus (NCBI Accession number: NP_734219.1) was submitted for 3-dimensional structural prediction using I-TASSER server [47]. I-TASSER identified structure of potyvirus VPg (PDB ID: 6NFB) as a template to generate the model of turnip mosaic virus VPg. The coordinates of the predicted models were refined using Galaxy Web (http://galaxy.seoklab.org/) [48]. The quality of the models was evaluated using procheck [49]. The coordinate of wheat eIFiso4E (PDB ID: 2IDR) [50] was downloaded and missing atoms were fixed manually using coot. The coordinates were further refined using Galaxy Web server. The refined coordinate was subjected for phosphorylation at serine 205 position using a post translational modification server, Vienna-PTM 2.0 [51]. Phosphorylated loop was refined again using the coot, before using it for the docking study. The modeled VPg structure was docked on eIFiso4E and phosphorylated eIFiso4E using HADDOCK 2.2 [52]. The interactions between putative complexes were visualized using Pymol [53].

### Statistical analysis

For all experimental assay measurements each sample was repeated at least three times, and the average value of three sets of experimental data is reported. The values of kinetic rate and data from CD experiments are represented as the mean ± SD (average standard deviation). Kaleida Graph software was used to fit the average set of data for least-squares fitting of the data with linear equations and determination of standard errors for parameters obtained from the fits.

## Results

**Fig 1:**
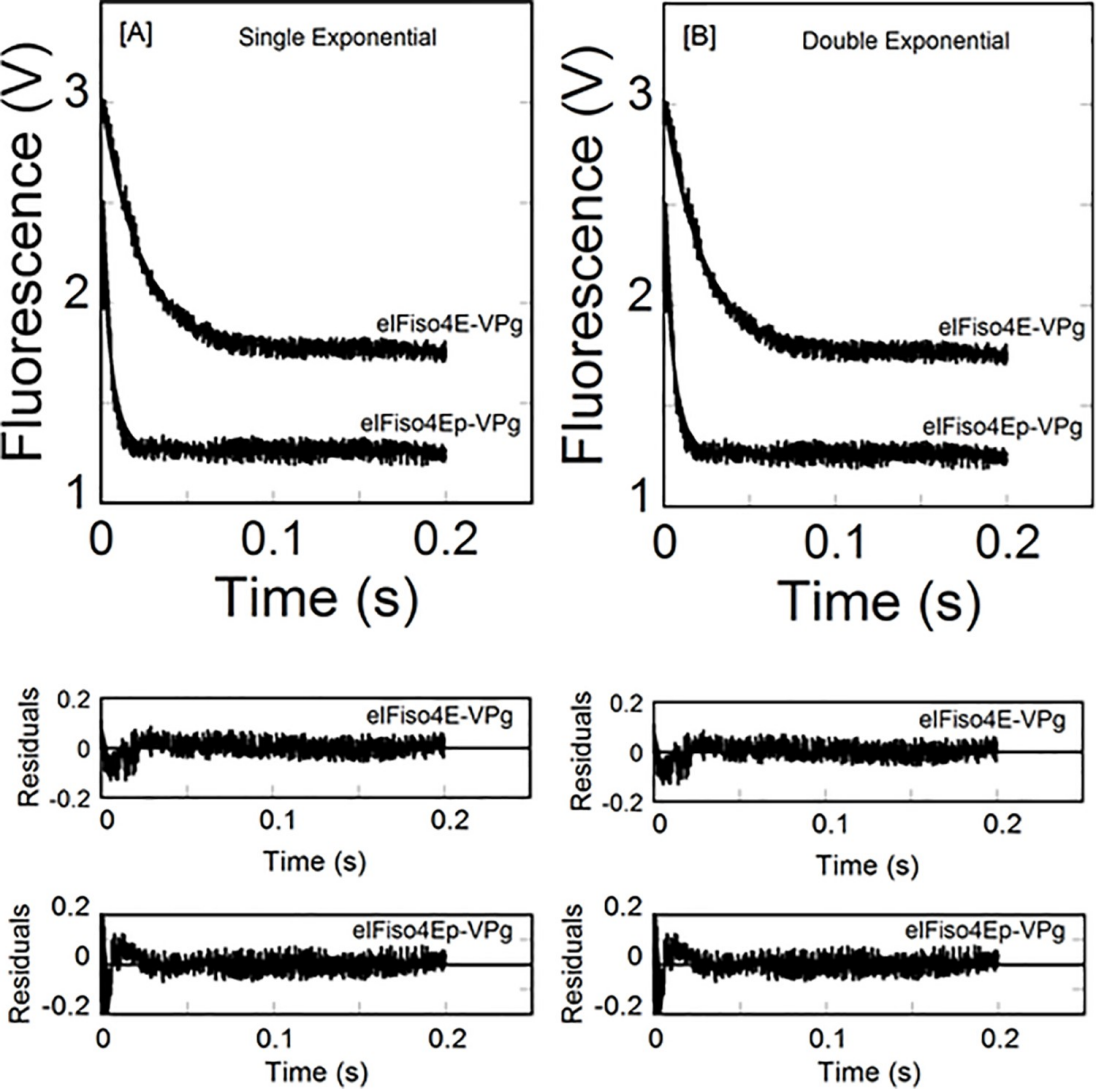
Phosphorylation enhances the kinetic rates for the binding of eIFiso4E with VPg. eIFiso4Ep or eIFiso4E (0.2 μM) in one syringe was mixed with VPg (2.0 μM) in the other syringe at 298 K. (A) Single-exponential fit to the kinetic data. (B) Double-exponential fit to the same kinetic data as in panel A. Solid lines represent the fitted curve drawn through the data points was best fit by assuming a single-exponential fit. Residuals for the corresponding fits are shown in the *lower panels*.

We have previously [36] shown the equilibrium binding of phosphorylated eIFiso4E with viral genome-linked protein VPg of turnip mosaic virus. Phosphorylation enhanced the binding affinity of eIFiso4E with VPg and activates *in vitro* translation. To examine the effect of phosphorylation on VPg-dependent viral translation, we further investigated the binding rates of eIFiso4Ep interaction to VPg of turnip mosaic virus by stopped-flow experiments. Fig 1 shows representative kinetic traces obtained upon rapid mixing of 0.2 μM eIFiso4Ep or eIFiso4E with 2.0 μM VPg (concentrations refer to those before mixing). Experiments were conducted using high concentration of VPg and limiting concentrations of eIFiso4Ep to ensure that the bimolecular combination of VPg and eIFiso4Ep was pseudo-first order. Stopped-flow data for a typical reaction of eIFiso4Ep binding to VPg were plotted as the fluorescence change (in volts) *versus* time (Fig 1). Time course data were fitted to a single- and double-exponential equation using nonlinear regression analysis [54] as described in materials and methods. The residuals representing the deviation between the calculated and experimental data indicate that the kinetic traces followed single-exponential fits over the time course measurements. Stopped flow fluorescence data show that the observed rate constant (*k*_obs_) of eIFiso4Ep is about 4-fold faster than eIFiso4E binding to VPg (eIFiso4Ep∙VPg, *k*_obs_ = 213 ± 11.3 s^-1^; eIFiso4E∙VPg, *k*_obs_ = 51 ± 3.4 s^-1^; Table 1) at 298 K. Analysis of the data using a double-exponential equation did not improve the fitting results (Fig 1B). The residuals (Fig 1, *lower panel*) neither varied over the time course nor diminished by a double-exponential fit.

**Table 1:** Kinetic parameters for the binding of phosphorylated and unphosphorylated eIFiso4E with VPg of turnip mosaic virus.

Complex	Observed rate constant, *k*_obs_ (s^–1^)					*E*_a_ *k* –_2_ x 10^3^	
	278 K	283 K	288 K	293 K	298 K	(kJ/mole) (s^–1^)	
eIFiso4Ep·VPg	41.4 ± 0.3	73.6 ± 3.5	98.7 ± 3.8	148.4 ± 9.8	213 ± 11.3	37.2 ± 2.8	13.2 ± 0.9
eIFiso4E·VPg	8.9 ± 0.7*	14 ± 0.6*	22 ± 0.5*	35 ± 0.9	51 ± 3.4	52.6 ± 3.6	37.6 ± 1.8

*Values taken from reference [3].

**Fig 2:**
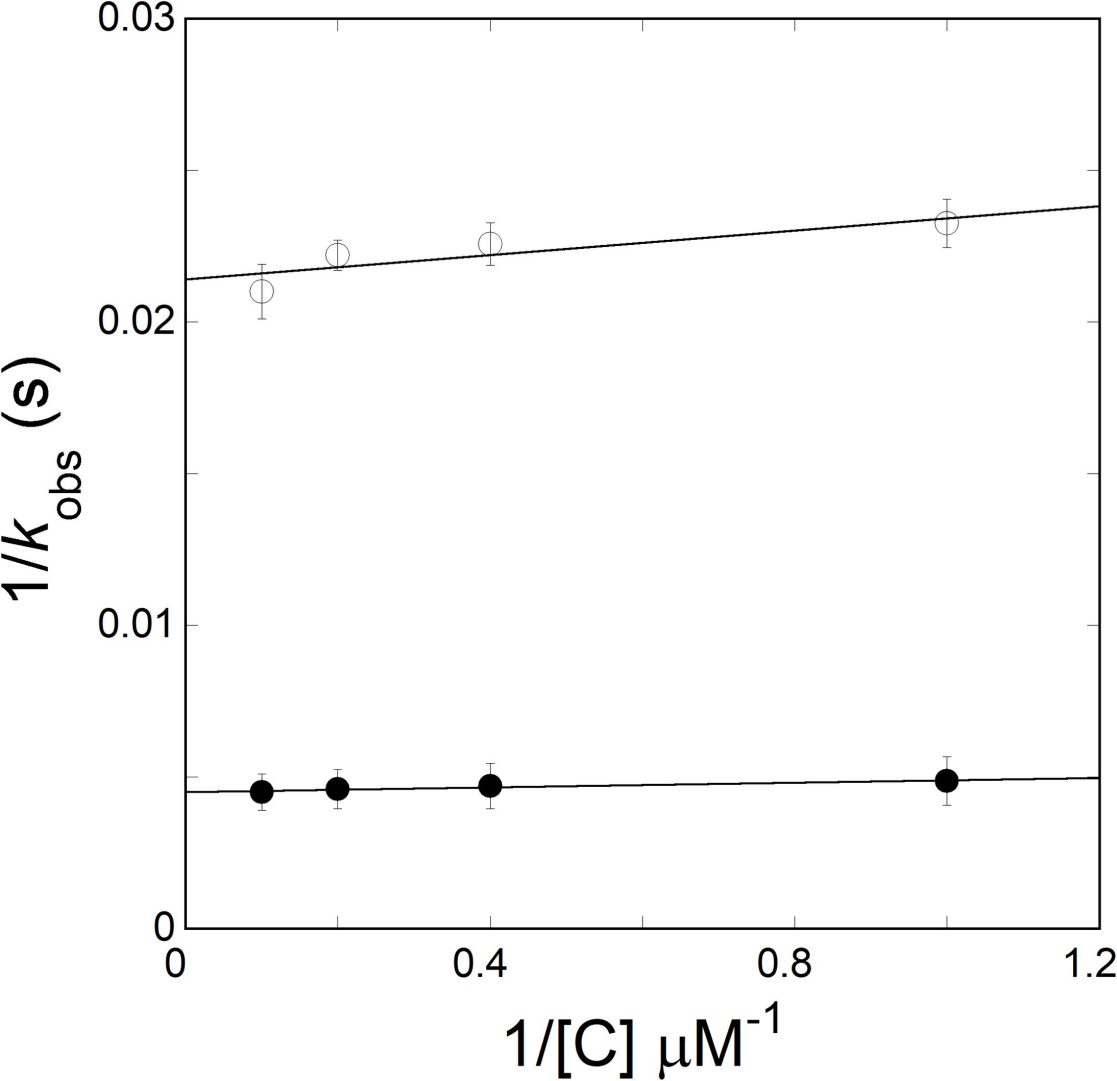
Dependence of *k*_obs_ on VPg concentration for reactions conducted with phosphorylated and unphosphorylated eIFiso4E. The value of *k*_2_ obtained for reaction of the mixture of 0.2 μM of eIFiso4Ep (—●—) or eIFiso4E (—О—) with VPg (1–10 μM) from the reciprocal of the y intercept of the plots of 1/*k*_obs_ *versus* 1/[C]. *k*_2_ was determined from a least-squares fit of the data to a linear function (*solid line*).


Under pseudo-first order conditions, where VPg was saturating with limiting concentration of eIFiso4Ep, [eIFiso4Ep] << [VPg], the observed rate constant was predicted to be a linear function of the VPg concentration. However, as observed previously [18], the results showed that the observed binding rates did not dependent on VPg concentration. Fig 2 shows the association plot of 1/*k*_obs_ *versus* 1/[VPg], featuring the *k*_obs_ of phosphorylated eIFiso4E was not depending on the VPg concentrations upto 50-fold excess of eIFiso4Ep. The observed rate varied from 208 to 219 s^-1^ for the eIFiso4Ep-VPg complex. As proposed previously [18], the binding mechanism of eIFiso4Ep-VPg complex can be explained by the following scheme:

$$\begin{array}{c} eIFiso4Ep\;+\;VPg\;\overset{K_{D}}{⇌}\;eIFiso4Ep∙VPg\\ {}[eIFiso4Ep∙VPg]\;\overset{k_{2}}{\to }\;eIFiso4Ep∙VPg \end{array}$$

The binding rates are related to the concentration of substrate as described previously [18, 54],

$$k_{\mathrm{obs}}\;=\;k_{2}\frac{\left[\mathrm{VPg}\right]}{K_{D}+[\mathrm{VPg}]}$$

where *k*_obs_ is the observed rate constant, *k*_2_ is the forward rate constant, K_D_ is the dissociation constant. Fig 2 shows 1*/k*_obs_ *versus* 1/[C] of VPg, which shows no significant dependence of *k*_obs_ on VPg concentration. *k*_2_ was determined from the intercepts of 1/[C] *versus* 1/*k*_obs_, to be 56.1 ± 3.7 s^-1^ and 227 ± 14.0 s^-1^, respectively, for eIFiso4E∙VPg and eIFiso4Ep∙VPg complexes.

**Fig 3:**
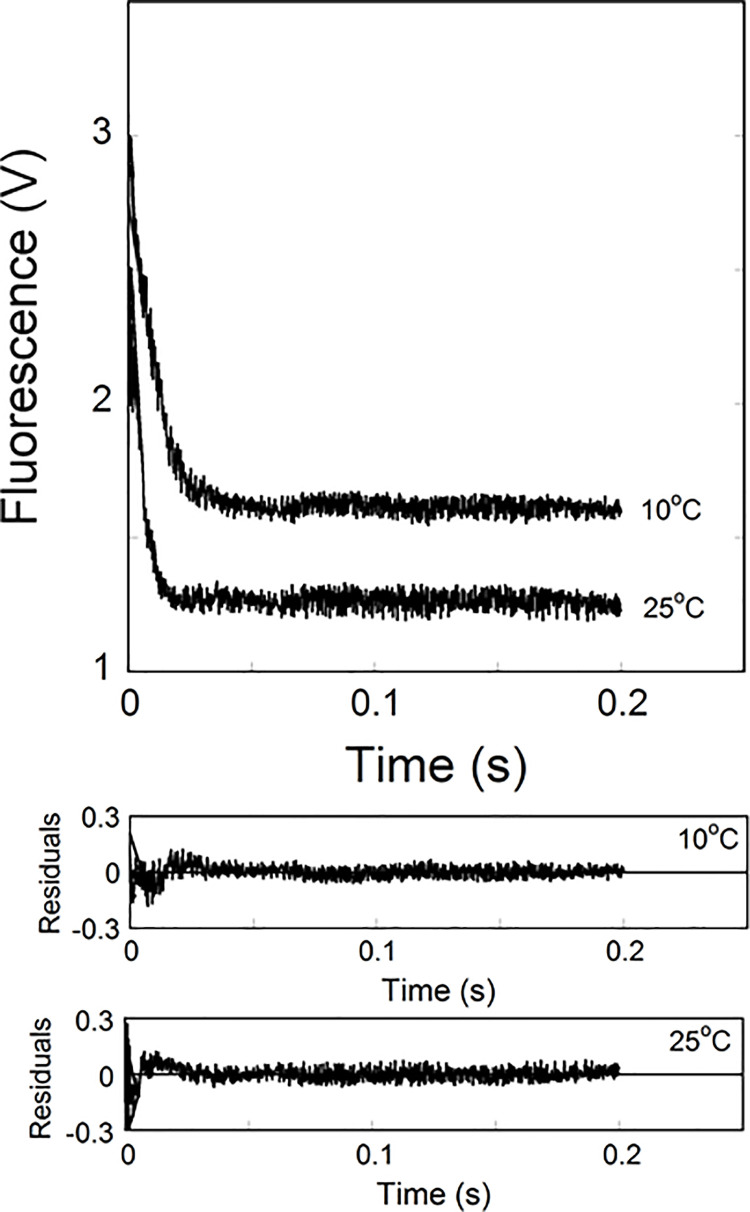
Temperature dependence Kinetic measurements of eIFiso4Ep∙VPg binding. Kinetic traces of eIFiso4Ep (0.1 μM, final) association with VPg (1.0 μM, final) at temperature 283 K and 298 K. The curve (*solid line*) represents the fitted line drawn through the data points was fit by assuming a single exponential process. Residuals for the fits are shown in the *lower panels*.

Temperature-dependent observed rate constant (*k*_obs_) was calculated from the data sets collected at each temperature for the binding of eIFiso4E and eIFiso4Ep with VPg. The *k*_obs_ values for the eIFiso4Ep∙VPg and eIFiso4E∙VPg complexes increased with an increase in temperature (Table 1, Fig 3). The stopped-flow kinetic data revealed that eIFiso4Ep∙VPg had the higher rate constants compared to the eIFiso4E∙VPg complex at all temperature. The rate constant of eIFiso4Ep∙VPg was 4-fold faster than eIFiso4E∙VPg at 298 K (Table 1). Fig 3 shows the temperature-dependent binding rates for the interaction of VPg with eIFiso4Ep. The kinetic data showed that eIFiso4Ep∙VPg complex binding rate (*k*_obs_ = 213 ± 11.3 s^-1^) at 298 K was ~6-fold faster as compared to 278 K (*k*_obs_ = 41.4 ± 1.6 s^-1^). This suggested that the rate of viral protein synthesis increased when plant hosts were grown at an elevated temperature.

**Fig 4:**
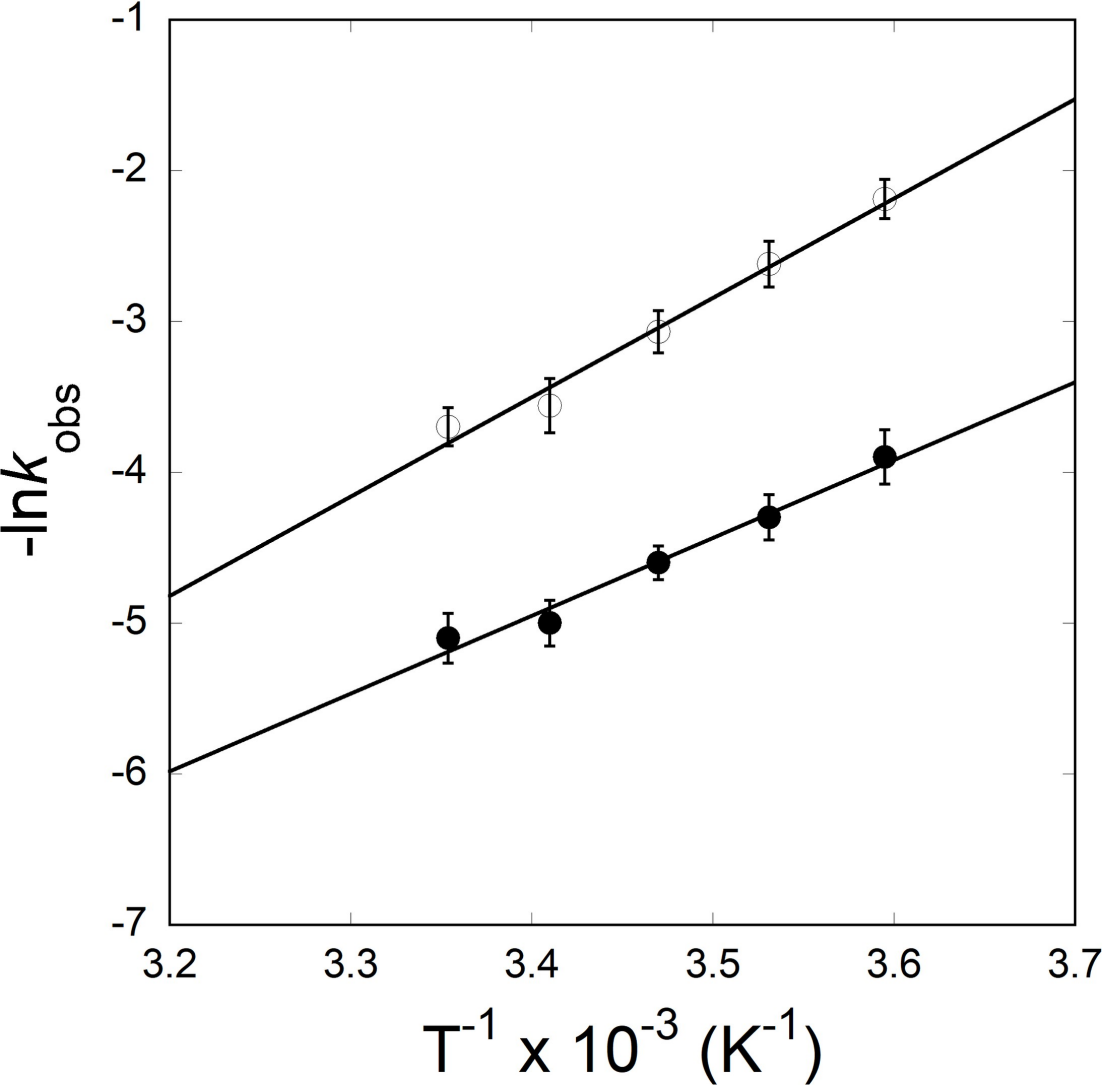
Arrhenius plots for the interaction of phosphorylated and unphosphorylated eIFiso4E with VPg. Dependence of *k*_obs_ on temperature (278–298 K) for reaction conducted for eIFiso4Ep (—●—) and eIFiso4E (—О—), with VPg. Data are fit with Eq 3. The value of *E*_a_ for the complexes were calculated from the slope of the fitted linear plot of ln *k*_*obs*_ versus 1/*T* (Kelvin) using least-squares fit of the data to a linear function.

To determine the activation energy of the eIFiso4Ep∙VPg complex, temperature-dependent observed rate constant values were used to construct an Arrhenius plot (Fig 4) according to Eq 3. The activation energies were calculated from the slopes of the linear fit of ln*k*_*obs*_ *versus* 1/T. The activation energies for the eIFiso4Ep∙VPg and eIFiso4E∙VPg complexes were 37.2 ± 2.8 kJ/mol and 52.6 ± 3.6 kJ/mol [3], respectively. These data show a large reduction in activation energy for eIFiso4Ep binding to VPg. The reduction in activation energy for eIFiso4Ep∙VPg complex suggests number of more favorable hydrogen bonds are formed in the transition state upon phosphorylation. The activation energy is related to the enthalpy of reaction [3], suggesting hydrogen bonds or ionic interactions rather than hydrophobic forces, which usually are entropically favorable, are involved [3]. The overall reduction in activation energy for eIFiso4Ep∙VPg complex suggests that phosphorylation succeeds in conformational change of the complex, allowing faster eIFiso4E binding and an increase in viral protein synthesis.

**Fig 5:**
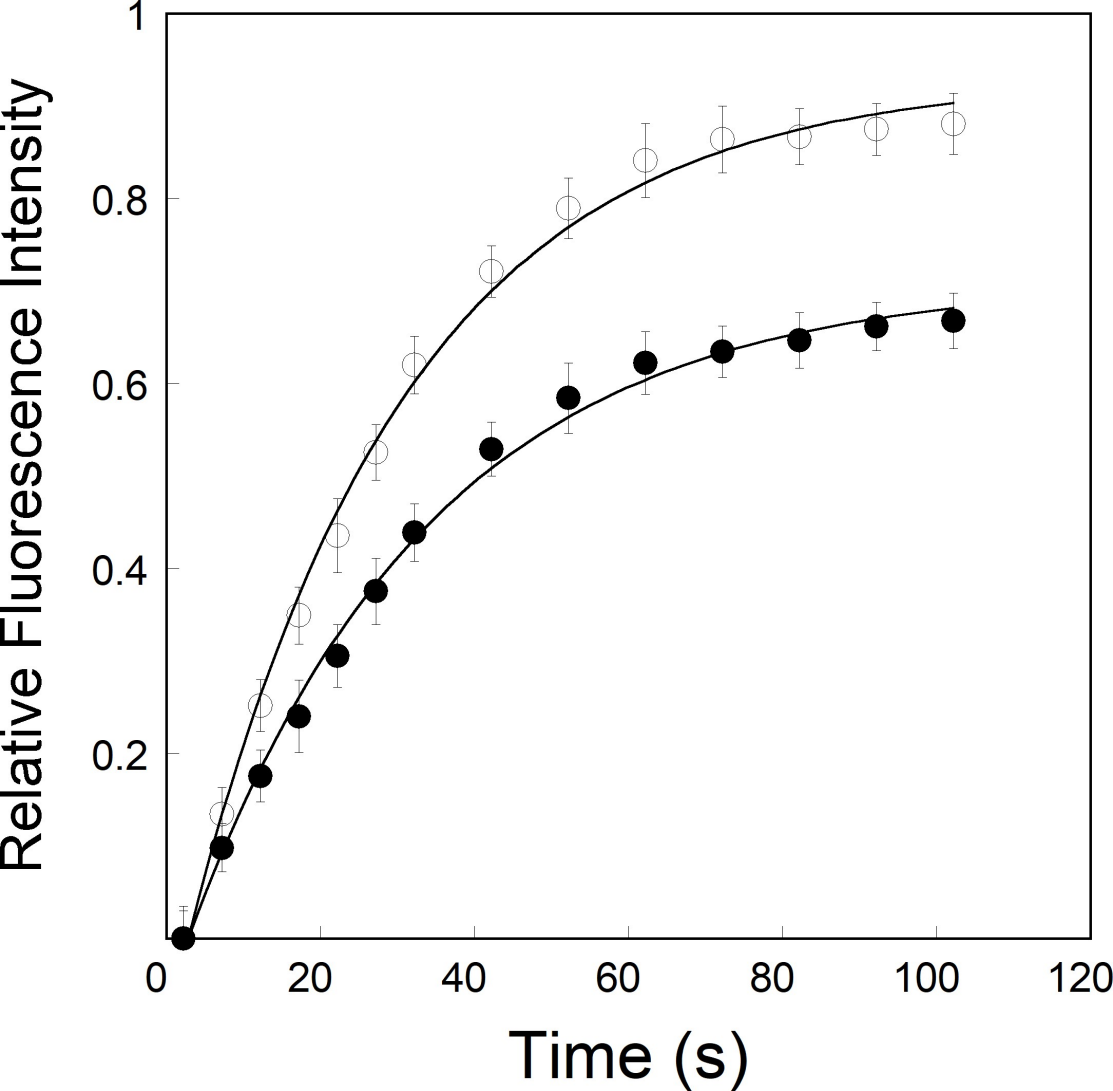
Dissociation kinetics of VPg from pre-formed eIFiso4Ep∙VPg complex. Dissociation rate (*k*_-2_) constant were monitored by rapidly diluting 100 μl of the complex eIFiso4Ep∙VPg (—●—) or eIFiso4E·VPg (—О—) with 900 μl of titration buffer at 298 K. VPg and eIFiso4Ep or eIFiso4E concentrations were 10 μM and 1 μM.

To test this reaction mechanism in detail, we investigated the effect of phosphorylation on the dissociation rates (*k*_-2_) of eIFiso4Ep∙VPg complex using fluorescence measurements. The dissociation rates for the eIFiso4Ep∙VPg and eIFiso4E∙VPg complexes were monitored by the dilution (10-fold) with buffer in the fluorescence cuvette. Fig 5 shows the results of the dilution experiment for the complexes. The dissociation rate constants for eIFiso4Ep∙VPg and eIFiso4E∙VPg complexes were obtained from the fitted curves as (13.2 ± 0.9) x 10^−3^ s^-1^ and (37.6 ± 1.8) x 10^−3^ s^-1^ (Table 1). Phosphorylation on eIFiso4E decreased the dissociation rate about 3-fold for VPg from eIFiso4E∙VPg. These results show that eIFiso4Ep binds to VPg more rapidly and dissociates more slowly as compared to eIFiso4E.

**Fig 6:**
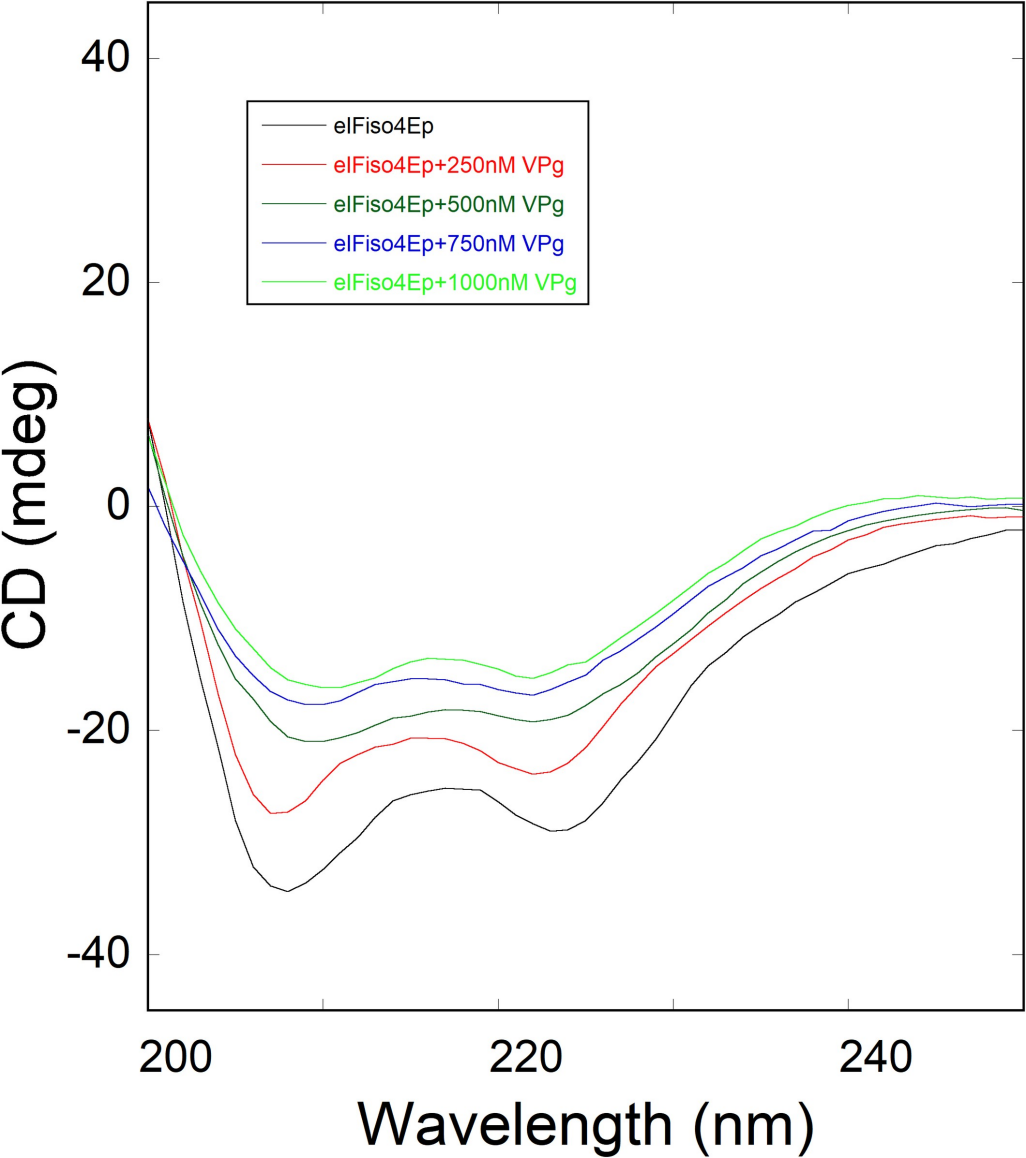
Far-UV CD spectra of phosphorylated eIFiso4E as a function of VPg. Far-UV CD spectra were obtained using eIFiso4Ep concentration of 0.1 μM with addition of varying concentrations of VPg (0–1000 nM).

To investigate the conformational changes in the eIFiso4Ep due to binding of VPg, the far-UV (200–250 nm) CD spectrum of eIFiso4Ep were collected. Circular dichroism measurements in the far-UV region yield data that has been used in analyzing the changes in the secondary structure of protein molecule, such as amount of α-helix, β-sheet, β-turn, and random coil [55, 56]. In this study, we measured the CD spectrum of eIFiso4Ep alone and then, the conformational changes of eIFiso4Ep resulted due to addition of VPg. Fig 6 shows the far UV CD spectra of eIFiso4Ep binding to VPg. EIFiso4Ep (0.1 μM), when treated with different amounts of VPg (0–1 μM), exhibited a change in minima at 208 nm in the CD spectrum of eIFiso4Ep in the far-UV region, reflecting a decrease in an α-helical content and an increased in β-sheet upon binding with VPg. The far-UV CD spectra of eIFiso4Ep protein are characterized by two minima at 208 and 222 nm [57] (Fig 6) representing the formation of an α-helical conformation [44, 58]. The flattening of the spectrum between the nearly equivalent minima at 208 and 222 nm is a characteristic feature found in protein of the α/β structural class that contain domains comprised of intermixed α-helical and β-sheet structures [59]. At the highest VPg concentration, eIFiso4Ep spectrum almost flattened at 208 and 222 nm, indicating that the structural elements present in the eIFiso4Ep have adopted conformations other than the dominant α-helix. At increasing concentrations of VPg to eIFiso4Ep produced significant alteration in the CD values at these wavelengths, indicating secondary structural change in eIFiso4Ep protein and suggested complex formation between eIFiso4Ep and VPg. Changes in the secondary structures content with addition of increasing amounts of VPg induce the less ordered α-helical structure of the eIFiso4Ep protein has folded into active beta-sheet conformation. The secondary structural contents were estimated as described previously [44, 60, 61]. Binding of VPg to eIFiso4Ep reduced the α-helix content to about 35% with an increase in the β-sheet content to about 25%. We observed decrease in the α-helicity values of eIFiso4Ep with addition of VPg. These changes in secondary structure reflect the eIFiso4Ep undergoes certain conformational changes due to addition of VPg. This large conformational transition suggests eIFiso4Ep binds to VPg by interaction of a β-sheet motif and that this conformational transition may have a regulatory role.

**Fig 7:**
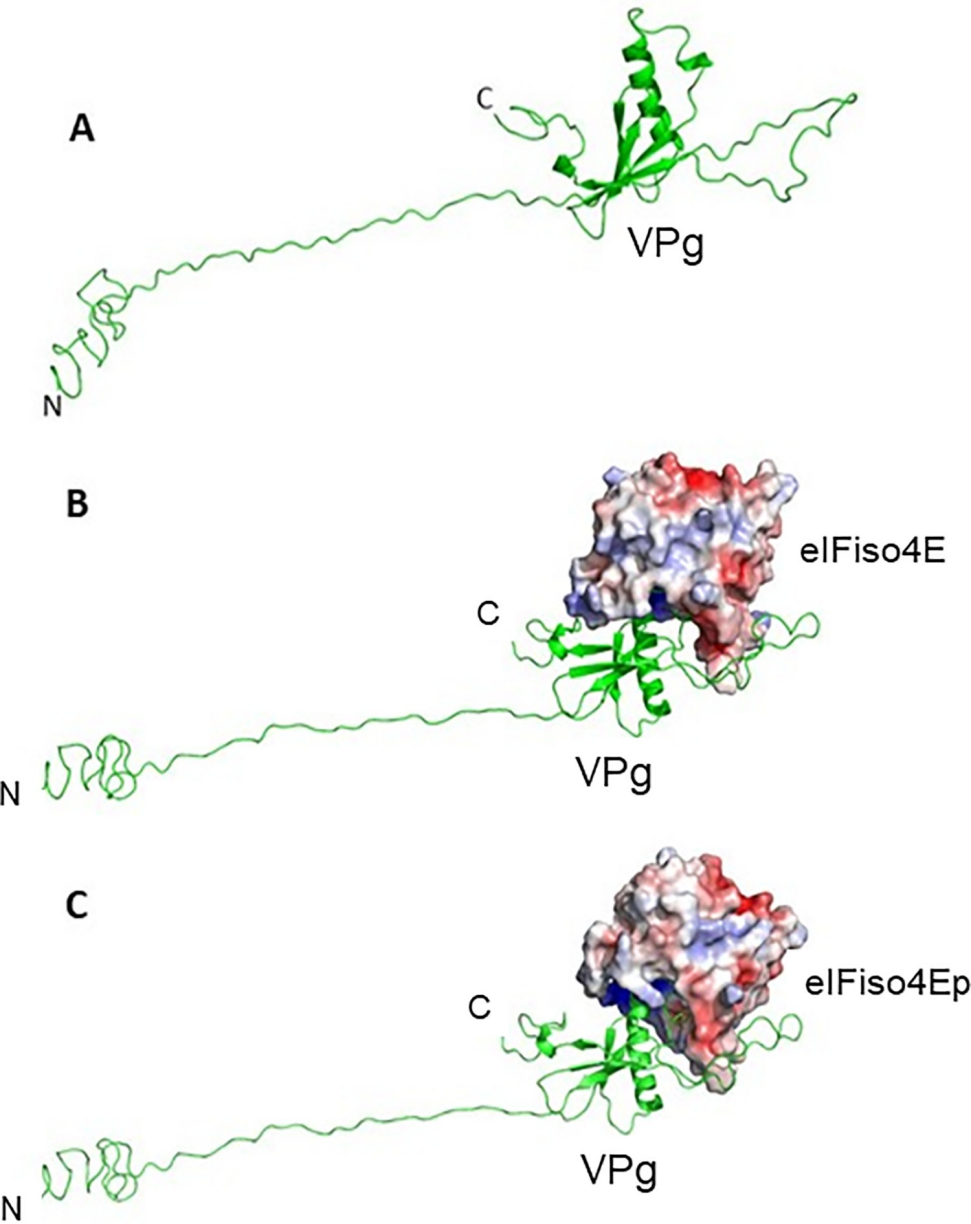
Molecular modeling of VPg and docking interactions with phosphorylated eIFiso4E. (A) Homology model of full-length turnip mosaic virus VPg shown in pale green cartoon representation. (B) Docking of VPg model (pale green cartoon) with eIFiso4E. (C) Docking of VPg model (pale green cartoon) with eIFiso4Ep. eIFiso4E and eIFiso4Ep are represented as surface electrostatic diagram.

**Fig 8:**
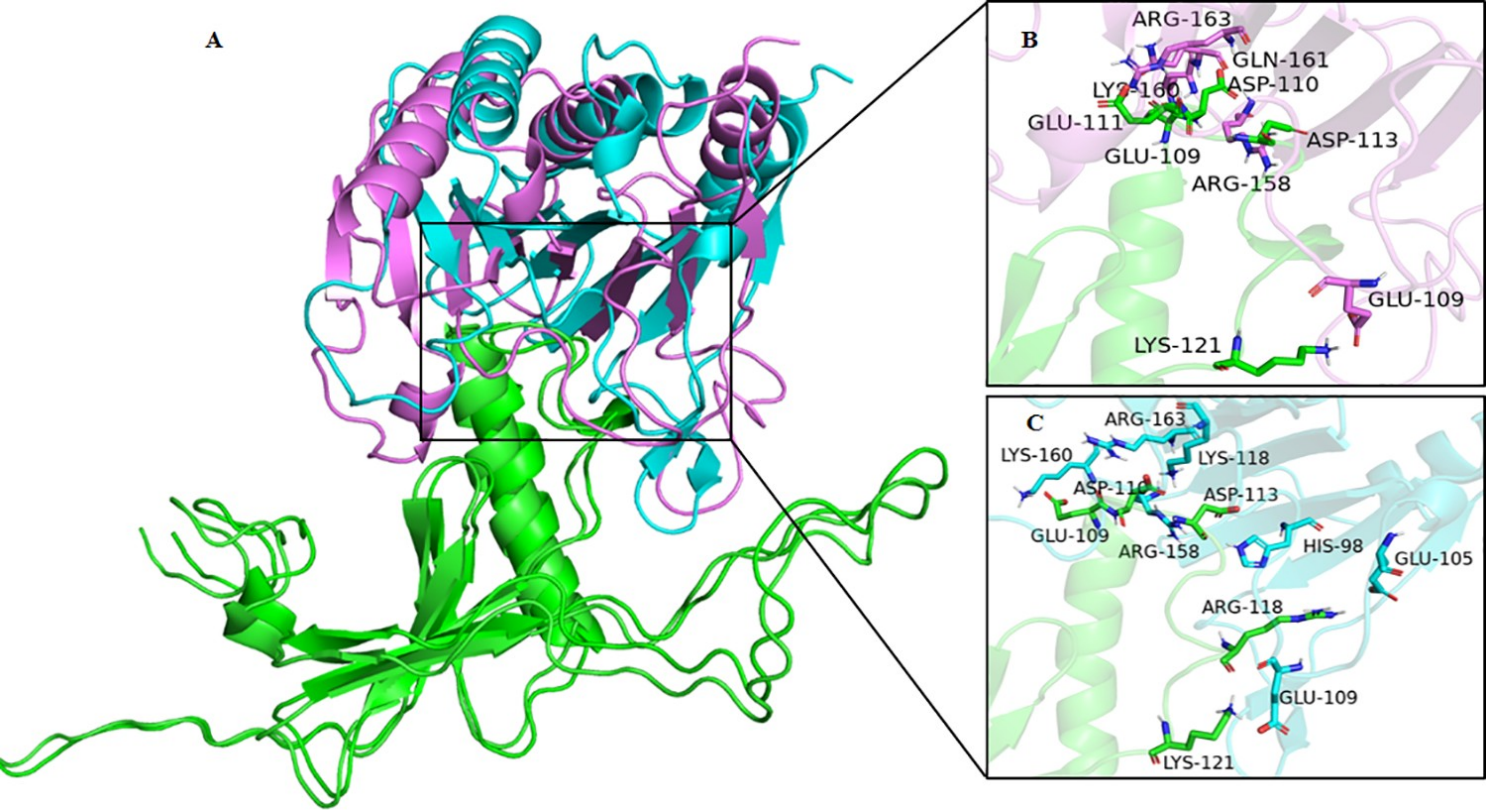
Representation of predicted mode of interaction of VPg with eIFiso4E and eIFiso4Ep. (A) Superimposition of docked VPg (pale green cartoon) with eIFiso4E (magenta cartoon) and eIFiso4Ep (cyan cartoon) complexes. (B) Interface residues between VPg and eIFioso4E. (C) Interface residues between VPg and eIFiso4Ep. Interface interaction residues are depicted as stick model with respective color codes.

The model of full-length turnip mosaic virus VPg was observed very similar to potyvirus VPg structure, where most of the N-terminal part and a stretch of C-terminal were unstructured. Like potyvirus VPg structure, only residues 73–178 of turnip mosaic virus VPg were generated ordered secondary structure components in the model. The structural part consisted of six beta-strands and one alpha-helix. The strand four and five were connected to a big loop (Fig 7A). Molecular docking models confirmed the binding of VPg with the eIFiso4E and eIFiso4Ep. In both the cases, the acidic residues of the VPg were observed to bind with a basic patch of the eIFiso4Ep (Fig 7B and 7C). Attachment of monophosphate at the serine 209 position of eIFiso4E did not induce any structural changes, except a subtle change in the loop region at the phosphorylation site was observed when compared with un-phosphorylated eIFiso4E. Comparison of docked VPg complexes with eIFiso4E and eIFiso4Ep showed a structural change in the eIFiso4Ep structure, suggesting that the binding of VPg induces a conformational change in eIFiso4Ep (Fig 8A). Analysis of interface residues by PDBePISA between VPg and eIFiso4E, revealed that apart from the common interacting residues, phosphorylated form was observed to participate with additional salt bridge interactions with the VPg (Fig 8B and 8C). Interacting residues from VPg and eIFiso4E are sited in S1 Table.

## Discussion

Phosphorylation plays an important role in the virus infectivity. Potyviruses RNA interactions to protein has been shown to be affected by phosphorylation. Protein synthesis is a dynamic process in which protein-protein or protein-RNA binding and release occur for each cycle of translation initiation. It has been demonstrated that potyviruses require host-cell translation machinery to replicate, and specifically, viral genome-linked protein VPg interacts with host plant eIF4E or its isoform eIFiso4E. This interaction is necessary for virus infectivity [6, 7, 18]. Although it had been recently shown that phosphorylation effects the binding of eIFiso4E with VPg, the kinetics of phosphorylated eIFiso4E with VPg had not been determined. We therefore compared the kinetic analysis of phosphorylated and unphosphorylated eIFiso4E binding to VPg of turnip mosaic virus. Using stopped flow measurements, we have shown that phosphorylation of eIFiso4E increased the binding rates about 4-fold for VPg, suggesting that phosphorylated protein was more able to accomplish the conformational change necessary for binding. Stopped flow investigations of the binding of eIF4E protein to VPg and m^7^G cap, under pseudo-first order reaction conditions, were performed as described by Walter *et al*. [18] and by Slepenkov *et al*. [62]. The first group concluded that when VPg form complex with eIF4E switches from a single step to a two-step kinetic model. However, according to the second group a one-step reaction is sufficient to describe the kinetic data of phosphorylated eIF4E with m^7^G cap. We investigated the association of eIFiso4E with VPg or cap analogues using stopped flow spectrofluorimetry under pseudo-first order reaction conditions [3, 63]. In the earlier study we have used single and double-exponential functions in experimental data fitting and the obtained rate constants were interpreted in terms of plausible one- and two-step association mechanisms. In the present study the mechanism of phosphorylated eIFiso4E association with VPg is consistent with the Michon and coworkers [18] kinetic mechanism for the interaction of eIF4E with VPg peptides. The kinetic rates for the interaction of eIFiso4Ep with VPg follow two-step binding mechanisms with the first step being fast association followed by a conformational change of the complex in a rather fast step, but with measurable rate. Kinetic data followed a single-exponential fit. A plot of 1/*k*_obs_ *versus* 1/[VPg] gave a straight line, showing the pseudo-first order character of the association depending on the VPg concentration. Phosphorylation increased the kinetic rates and decreased the dissociation rate about 4- and 3-fold for the eIFiso4E binding to VPg. A significant difference in the association and dissociation rate is seen upon the phosphorylation of eIFiso4E binding with VPg. These data favor the formation of eIFiso4Ep∙VPg stable complex, and the rates indicate a mechanism where VPg could kinetically compete with cap for eIF4E or its isoform eIFiso4E binding [3, 17]. Moreover, the kinetic rates for the binding of eIFiso4Ep with VPg (*k*_obs_ = 148.4 s^-1^, Table 1) is faster than the binding rate of m^7^G cap for eIFiso4E (*k*_obs_ = 30.6 s^-1^) [64]. These data suggest that VPg is bound faster than the m^7^G cap binding to eIFiso4E and may function similarly to the 5’-cap structure of eukaryotic mRNA. The binding rates for the eIFiso4Ep was about 4-fold faster for VPg, suggesting that phosphorylated protein was more able to accomplish the conformational change necessary for binding. The phosphorylation of eIFiso4E enhances the binding of eIFiso4E∙VPg and mRNA translation *in vitro* [39]. This fast reaction of eIFiso4E with VPg upon phosphorylation suggest that VPg more rapidly forms a stable complex with eIFiso4E. EIFiso4E∙VPg complex significantly lowered the activation energy suggest that phosphorylation succeeds in speeding up reaction by providing a path with substantial lower energy barrier for efficient viral translation. It was reported that binding of eIF4E with VPg promotes a conformational change to proteins behavior of a pre-molten globule and leads to enhanced association which is necessary for the infectious ability *in vivo* [29]. It was suggested that VPg from lettuce mosaic virus strongly binds to the lettuce eIF4E and enhances lettuce mosaic virus infectious ability *in vivo* [18, 29]. Furthermore, conformational change of the VPg from potato virus has been confirmed by 3-dimentional structure and characterized the complex with eIF4E [17]. It has been reported that phosphorylation affects the conformational change of eIFiso4F [59]. The phosphorylation of eIFiso4E enhances the eIFiso4E∙VPg binding via conformational changes similar to those observed for cap binding [65]. The spectra of eIFiso4Ep are characterized by the appearance of two minima at 208 nm and 222 nm, representing the presence of an α-helical content in the protein. Furthermore, we observed that VPg induced significant change in α-helical content and beta-sheet content suggesting secondary structural change in eIFiso4Ep. Such structural change may result in increased specificity for VPg binding to eIFiso4Ep, which was further supported by the structural behavior change occurs upon the formation of eIF4E∙VPg complexes, which implicates the viral translation [17]. The observed change in kinetic parameters supports the structural changes in the eIFiso4Ep upon binding with VPg. These results were further supported by molecular docking studies, which shows that eIFiso4Ep adopts a conformational change upon binding with VPg (Fig 8A). Moreover, the conformational change generates additional residues interacting at the docking interface between VPg and eIFiso4Ep, suggests that the phosphorylation of eIFiso4E could induce a better binding with VPg. This conformational change could bring other initiation factors in close proximity to stimulate RNA circularization and VPg could inhibit endogenous mRNA translation [66] which leads to efficient viral translation.

## Supporting information

S1 Table Interaction interface residues. (DOCX)

S1 Data Dependence of *K*_obs_ on VPg concentration for reactions conducted with phosphorylated and unphosphorylated eIFiso4E. (XLSX)

S2 Data Arrhenius plots for the interaction of phosphorylated and unphosphorylated eIFiso4E with VPg. (XLSX)

S3 Data Dissociation kinetics of VPg from pre-formed eIFiso4Ep∙VPg complex. (XLSX)
