# Supplementary material for: Phosphorylation of eukaryotic initiation factor eIFiso4E enhances the binding rates to VPg of turnip mosaic virus
Source: PLoS One. 2021 Nov 4;16(11):e0259688. doi: 10.1371/journal.pone.0259688 (PMC8568277; doi:10.1371/journal.pone.0259688)
Supplement: S1 Table — (DOCX) [file pone.0259688.s001.docx]

**S1 Table: Interaction interface residues**

| Phosphorylated eIFiso4E residues | VPg residues |
| --- | --- |
| W49, Q59, K56, R58, G63, S64, T65, I66, H67, S89, K90, L91, N92, V93, D96, H98, F100, P106, K107, W108, E109, D110, I112, K118, S156, V157, R158, Q159, H199, E200, A202, K203, R204, S205, D206, K207, P209 | R7, K11, R22, E23, Y25, L79, T80, G81, A82, T83, I102, M104, D105, L106, L107, G108, E109, E111, L112, D113, S114, N115, E116, R118, K121, N183, E185, L186, V187, D190 |
| Un-phosphorylated eIFiso4E residues | VPg residues |
| W49, Q59, W62, G63, S64, T65, H67, D96, F97, H98, F100, P106, K107, W108, E109, D110, P111, I112, K118, V154, V155, S156, R158, R163, W167, H199, K203, S205, D206, K207 | K4, R5, Q8, V24, Y25, G26, I31, L79, T80, G81, M104, D105, L107, G108, E109, D110, E111, L112, D113, S114, N115, E116, R118, I144, L146, E162, N183, L186 |
